# Total marrow irradiation reduces organ damage and enhances tissue repair with the potential to increase the targeted dose of bone marrow in both young and old mice

**DOI:** 10.3389/fonc.2022.1045016

**Published:** 2022-11-10

**Authors:** Ji Eun Lim, Srideshikan Sargur Madabushi, Paresh Vishwasrao, Joo Y. Song, Amr M. H. Abdelhamid, Hemendra Ghimire, V. L. Vanishree, Jatinder K. Lamba, Savita Dandapani, Amandeep Salhotra, Mengistu Lemecha, Antonio Pierini, Daohong Zhao, Guy Storme, Shernan Holtan, Cynthia Aristei, Dorthe Schaue, Monzr Al Malki, Susanta K. Hui

**Affiliations:** ^1^ Department of Radiation Oncology, City of Hope National Medical Center, Duarte, CA, United States; ^2^ Department of Pathology, City of Hope Comprehensive Cancer Center, Duarte, CA, United States; ^3^ Radiation Oncology Section, Department of Medicine and Surgery, Perugia University and General Hospital, Perugia, Italy; ^4^ Department of Oncology and Nuclear Medicine, Faculty of Medicine, Ain Shams University, Cairo, Egypt; ^5^ Department of Pharmacotherapy and Translational Research, College of Pharmacy, University of Florida, Gianesville, FL, United States; ^6^ Department of Hematology and Hematopoietic Cell Transplantation, City of Hope National Medical Center, Duarte, CA, United States; ^7^ Department of Molecular and Cellular Biology, Beckman Research Institute, Duarte, CA, United States; ^8^ Division of Hematology and Bone Marrow Transplantation, Perugia General Hospital, Perugia, Italy; ^9^ Department of Biochemistry and Structural Biology, Univeristy of Texas (UT) Health San Antonio, San Antonio, TX, United States; ^10^ Department of Radiotherapy Universitair Ziekenhuis (UZ) Brussels, Brussels, Belgium; ^11^ Blood and Marrow Transplant Program, Department of Medicine, University of Minnesota, Minneapolis, MN, United States; ^12^ Department of Radiation Oncology, University of California, Los Angeles (UCLA), Los Angeles, CA, United States

**Keywords:** total marrow irradiation, bone marrow transplantation, aging, tissue damage, tissue repair, DNA damage

## Abstract

Total body irradiation (TBI) is a commonly used conditioning regimen for hematopoietic stem cell transplant (HCT), but dose heterogeneity and long-term organ toxicity pose significant challenges. Total marrow irradiation (TMI), an evolving radiation conditioning regimen for HCT can overcome the limitations of TBI by delivering the prescribed dose targeted to the bone marrow (BM) while sparing organs at risk. Recently, our group demonstrated that TMI up to 20 Gy in relapsed/refractory AML patients was feasible and efficacious, significantly improving 2-year overall survival compared to the standard treatment. Whether such dose escalation is feasible in elderly patients, and how the organ toxicity profile changes when switching to TMI in patients of all ages are critical questions that need to be addressed. We used our recently developed 3D image-guided preclinical TMI model and evaluated the radiation damage and its repair in key dose-limiting organs in young (~8 weeks) and old (~90 weeks) mice undergoing congenic bone marrow transplant (BMT). Engraftment was similar in both TMI and TBI-treated young and old mice. Dose escalation using TMI (12 to 16 Gy in two fractions) was well tolerated in mice of both age groups (90% survival ~12 Weeks post-BMT). In contrast, TBI at the higher dose of 16 Gy was particularly lethal in younger mice (0% survival ~2 weeks post-BMT) while old mice showed much more tolerance (75% survival ~13 weeks post-BMT) suggesting higher radio-resistance in aged organs. Histopathology confirmed worse acute and chronic organ damage in mice treated with TBI than TMI. As the damage was alleviated, the repair processes were augmented in the TMI-treated mice over TBI as measured by average villus height and a reduced ratio of relative mRNA levels of amphiregulin/epidermal growth factor (*areg*/*egf*). These findings suggest that organ sparing using TMI does not limit donor engraftment but significantly reduces normal tissue damage and preserves repair capacity with the potential for dose escalation in elderly patients.

## Introduction

Total body irradiation (TBI) has been a standard component of the conditioning regimen for hematopoietic stem cell transplantation (HSCT) for hematological malignancies (1–3). The success of HSCT is often determined by a balance of providing adequate conditioning for engraftment and eradicating residual cancerous cells versus organ toxicity from conditioning and the subsequent risk of graft-versus-host disease. Previous research has shown that increasing the TBI dose (12 Gy to 15.75 Gy) in patients with high risk of relapse reduced leukemia relapse, although there was no survival benefit because of treatment-related mortality due to radiation toxicity to organs (4). To reduce the irradiation toxicity in the vital organs and to increase targeting of residual cancer cells, computed tomography (CT) image-guided total marrow irradiation (TMI) was developed and translated for clinical studies (5–7). Several research groups have used the TMI conditioning regimen to improve the outcome of patients with leukemia in HSCT (8–10). Our recent success with clinical TMI suggests that targeted marrow radiation is feasible and improves survival by decreasing both toxicity and relapse (11, 12). Furthermore, we recently initiated a dose escalation study in the older patient population (>55 years) to expand the HCT for patients with relapsed/refractory AML (CTN # NCT03494569). Determination of how the toxicity profile will change between TBI to TMI and whether dose escalation will be feasible in older patients is an unmet need.

TBI with or without chemotherapy prior to HCT has short-term and long-term treatment-related toxicities including acute and chronic GVHD, pneumonitis, mucositis, diarrhea, cardiac dysfunction, hypothyroidism and chronic kidney disease (13–18). Efforts to reduce TBI toxicities include hypo/hyper fractionation, dose rate, shielding organs at risk (OAR) and conformal Intensity modulated techniques to give higher doses to the target volume while sparing doses to surrounding tissues (19–26). Although these efforts provide some protection to OAR and improve treatment related toxicities, long-term toxicities are still a major concern. TMI is a compelling alternative as it provides an opportunity for dose escalation towards enhanced leukemia cell killing without severe tissue/organ adverse effects observed with TBI. This is particularly crucial for older patients who cannot receive myeloablative conditioning, resulting in increased relapse and graft failure risks. Therefore, dose escalation using TMI in the elderly offers a real chance to enhance the therapeutic ratio and addresses an urgent clinical need. However, a comparative evaluation of organ toxicity, particularly for dose escalation, between TMI and TBI cannot be achieved in the clinic because of increased treatment-related mortality reported earlier (4).

Previous research has indicated several mechanisms by which TMI may prove more beneficial than TBI as a conditioning platform (27). We previously evaluated dosimetric and biological differences of TMI versus TBI in rodents early point after irradiation (28). Our first-generation film-based preclinical 2D TMI model provided limited dosimetric information for vital organs, thereby limiting mechanistic understanding of tissue damage from TMI or TBI. To overcome this limitation, we recently developed a three-dimensional multimodal image-guided TMI model for preclinical mouse study, which provided organ-specific quantitative dosimetry (dose volume histogram) and successfully used this TMI technique in BMT model (29).

The next critical step is an assessment of how TBI versus TMI causes changes in vital organ tissue damage and repair mechanisms. Previous studies suggest amphiregulin (AREG), the epidermal growth factor (EGF) receptor ligand has a critical role in organ development and promotes tissue repair under inflammatory conditions (30–32). Additionally, circulating AREG is elevated at the onset of acute and life-threatening GVHD (33) a major treatment related toxicity after radiation conditioning, and it portends poor prognosis. It is found during states of unresolved tissue damage, particularly if markedly elevated *relative* to EGF (34). In addition, AREG is activated in type 2 immune response and inflammatory lesions (34–36). Here we demonstrate that AREG/EGF levels correlate with damage/repair processes in organs post radiation and BMT in mice, furthering our ability to distinguish between TBI versus TMI in the context of gastrointestinal resistance and the effects of aging.

## Materials and method

### Animals

Animal studies were approved by the Institutional Animal Care and Use Committee at the City of Hope, National Medical Center, Duarte, CA. The C57BL/6J mice (JAX 000664) and B6.SJL-Ptprc^a^ Pepc^b^/BoyJ (JAX 002014) were purchased from Jackson laboratory, Maine, USA, and housed at the COH animal facility.

### TMI treatment plan

The image guide-TMI treatment strategy was developed as described (29). Both young and old mice were irradiated with TMI/TBI (2 fractions 24h apart) at -2 and -1 day before BMT. The radiation treatment used in this study: TMI (12:4), TBI (12:12), TMI (16:4), and TBI (16:16), with the first value indicating the dose delivered to the bone marrow and spleen and the second value indicating the dose to all other organs. Radiation beam layout of TBI and TMI by regions (beam size, isocenter location, normalization point) is provided in supplementary figure S1 and supplement table 1 [modified **Supplementary Table 1** of (29)]. For TBI planning, mouse CBCT scans were divided into 3 regions for treatment optimization, and parallel opposed beams with a beam size of 40 mm square collimators were used to create a homogenized dose within the center of the beams in each region. For TMI planning, mouse CT scans were divided into 7 regions for treatment optimization. Beam sizes varied (40 x 40mm to 5mm square or circle) for different regions using different collimator settings. **Supplementary Figure S1C**, shows a parallel opposed radiation beam width (green) covering the spine. In addition, the prescribed dose to the head including skull and oral cavity was maintained at 12 Gy for all TMI treatment plans, to prevent increased toxicities like mucositis in dose escalated treatments.

### Congenic bone marrow transplantation and engraftment

A congenic BMT was carried out by transplanting donor (CD45.1) BM cells into irradiated recipient (CD45.2) mice. The recipient mice were irradiated with TMI/TBI (2 fractions, 24 h apart) and transplanted with donor BM cells 24 h post radiation. Donor Bone marrow (BM) were harvested from femur and tibiae of young donor mice (8 weeks old, B6.SJL-Ptprc^a^ Pepc^b^/BoyJ and a total of 25 million whole BM cells were injected into irradiated recipient mice (8 weeks young or 90 weeks old mice, CD45.2, C57BL/6j, JAX 000664) for BMT. To check donor engraftment, peripheral blood was collected from the tail vein and cells were stained with Percp-Cy5.5 anti-CD45.1 antibody (#110728, Biolegend) and APC anti-CD45.2 antibody (#109814, Biolegend) after RBC lysis with ACK (#A1049201, Thermo scientific). Donor chimerism was analyzed by flow cytometry using a BD LSR Fortessa (BD Biosciences, San Jose, CA) with data analysis using FlowJo V10.1 software.

### H&E and trichrome staining

Tissue damage was estimated at 12 weeks after Rx/BMT by H&E staining including aged-matched unirradiated controls. Liver, gut, and lung tissues were collected and fixed with 10% Neutral Buffered Formalin for 2 days. Specifically, for gut harvest, 1 cm of jejunum (~14 cm apart from the stomach) were collected. Formalin-fixed, paraffin embedded tissues (FFPET) were cut at 3-4-micron sections and stained with hematoxylin and eosin (H&E) for morphologic evaluation. Villus height was quantified using Image J (NIH).

FFPET sections were also stained with a Trichrome kit to determine the extent of fibrosis in the lung, liver and gut according to the manufacturer’s recommended protocol (StatLab, Cat# KTMTR2LT).

### Immunofluorescence staining

Tissues were fixed in 10% neutral buffered formalin (NBF) for 1-2 days and incubated in 30% sucrose in PBS for 2 days at 4°C and then embedded in OCT compound. Frozen sections were used for immunofluorescence staining. To detect γ-H2AX Phospho-Histone H2AX (Ser139) (#2577, Cell signaling, USA), and FITC conjugated anti-Rabbit secondary antibody were used. Phalloidin-Fluor 594 (ab176757, Abcam, USA) was used for F-actin staining. Cells were mounted with Vectashield mounting medium containing DAPI (Vector Laboratories, USA) and scanned under a Zeiss LSM 900 confocal microscope (Carl Zeiss). Cell counts of γH2AX^+^ and total cells were measured by ImageJ.

### qPCR analysis

Tissue mRNA was extracted using the RNeasy mini kit (Qiagen, #74106, USA). For cDNA synthesis, 1 µg mRNA was reverse transcribed using the high capacity cDNA reverse transcription kits (Life technologies #4369913, USA). Real time PCR was proceeded with Taqman Fast Advanced Master Mix (Thermo Scientific #4444965) according to the manufacturer’s instructions. The following Taqman probes were used for qRT-PCR: Taqman probes Mouse Amphiregulin (Mm00437583_m1), Taqman probes Mouse EGF (Mm00438696_m1), and Taqman probes Mouse GAPDH (Mm99999915_g1). All reactions were run in duplicate with 45 cycles, on a Quant Studio 3 (Applied Biosystems, by Thermo Scientific, USA). qPCR cycle: Initial denaturation 95°C, 20 s; 45 cycles 95°C, 1 s; 60°C. 20s. ∆Ct method was used for the calculation of target gene expression by normalizing to the housekeeping gene, GAPDH.

### Statistics

All data are presented as Means ± SEM values. Statistical analyses were conducted using Prism (GraphPad) software. The unpaired Student’s t-test, Log-rank (Mantel-Cox) test, and Two-way ANOVA test were applied for testing at a 5% level of significance (*P-value < 0.05).

## Result

### TMI treatment plan maintains prescription dose to bone marrow while reducing doses to vital organs

**Figure 1:**
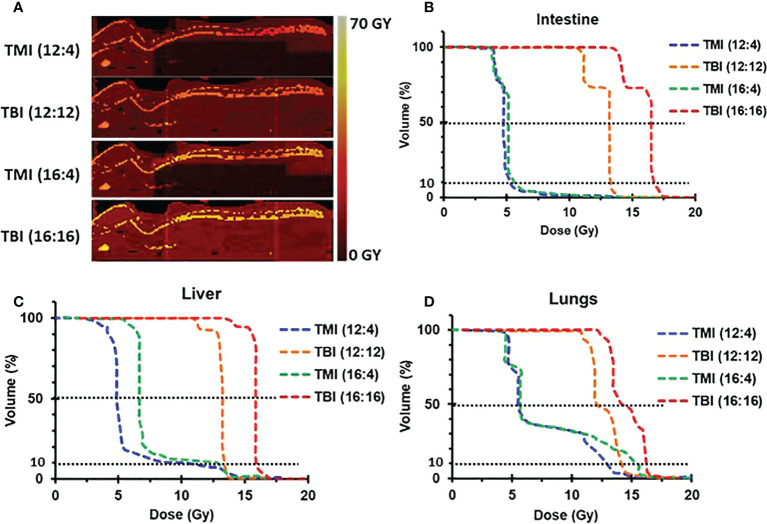
TMI vs. TBI strategy. Dose distribution for different radiation treatment regimens. **(A)** Radiation dose distribution of imaged-guided TMI or TBI strategy. Color painting of radiation dose 0-60 Gy. TMI showed 40-75% less radiation dose in liver, lung, and gut than TBI. **(B)** Dose volume histogram (DVH) of intestine. **(C)** DVH of liver. **(D)** DVH of lungs.

One of the most important advantages of our image guided TMI set-up is the ability to control the radiation dose to all organs, allowing us to precisely deliver BM treatment at a reduced dose to vital organs such as liver, lung, gut, and kidney, i.e. limiting normal tissue damage. Using this system, we have previously shown that TMI (12:4) is myeloablative in young mice (29) with further dose escalation feasible to 16 Gy BM dose, a 33% increase in dose, without changing the dose to the rest of the body which was maintained at 4 Gy, i.e. 16:4. Dose painting clearly indicates that the radiation dose in TMI is reduced in comparison to TBI which receives 100% of prescribed doses (**Figure 1A**). The mean organ dose to 50% volume (D50) is significantly reduced in all vital organs following TMI, be it in the (12:4) setting or at (16:4). For instance, there is 55-60% less dose to lung, 36-40% less to kidney, 55-60% less to liver, and the GI dose dropped by 55-60%, compared to TBI (~100% dose to all organs) (**Figures 1B–D**). The D10 (dose cover 10% volume) for liver, lung and kidney was ~80-90% of the prescribed dose, whereas D10 for GI was about 50% of the prescription dose suggesting that ~10% volume of lung, kidney and liver must have been near the planning target volume (PTV) i.e bone marrow. Importantly, the radiation dose to the BM was similar between TMI and TBI. The DVH of different organs is shown (**Table 1**). Overall, this supports the notion that compared to TBI, TMI treatment planning significantly reduces normal tissue exposure.

**Table 1:** Dose Distribution for different radiation treatment.

Regimens	TMI (12:4)		TBI (12:12)		TMI (16:4)		TBI (16:16)	
Dase stat	D50 (Gy)	D10 (Gy)	D50 (Gy)	D10 (Gy)	D50 (Gy)	D10 (Gy)	D50 (Gy)	D10 (Gy)
Intestine	4.8	5	11.9	12.6	5.2	5.4	15.5	16.4
Lungs	6.7	11.3	11.4	12.6	8.1	14.9	14.9	16.4
Liver	5.5	9.3	12.3	12.6	6.2	12	16	16.4
Kidneys	7.75	10.2	12.4	12.75	9.55	13.2	16.15	16.65
Spleen	12.9	17.8	12.7	12.9	17.2	Z4.6	16.5	16.8
PTV (Bones)	31.6	35.8	32	36.8	42.2	47.9	42.5	48.7

Dosimetry of PTV (Bones) and vital organs (Liver, Lung, Kidney, Intestine, spleen) of TMI (12:4), (16:4) and TBI (12:12), (16:16). D50 = mean radiation dose covering 50% of tissue volume, D10 = highest radiation dose covering 50% of tissue volume. D10 = highest radiation dose covering 10% of tissue volume.

### TMI based dose escalation is well tolerated in both young and old mice

**Figure 2:**
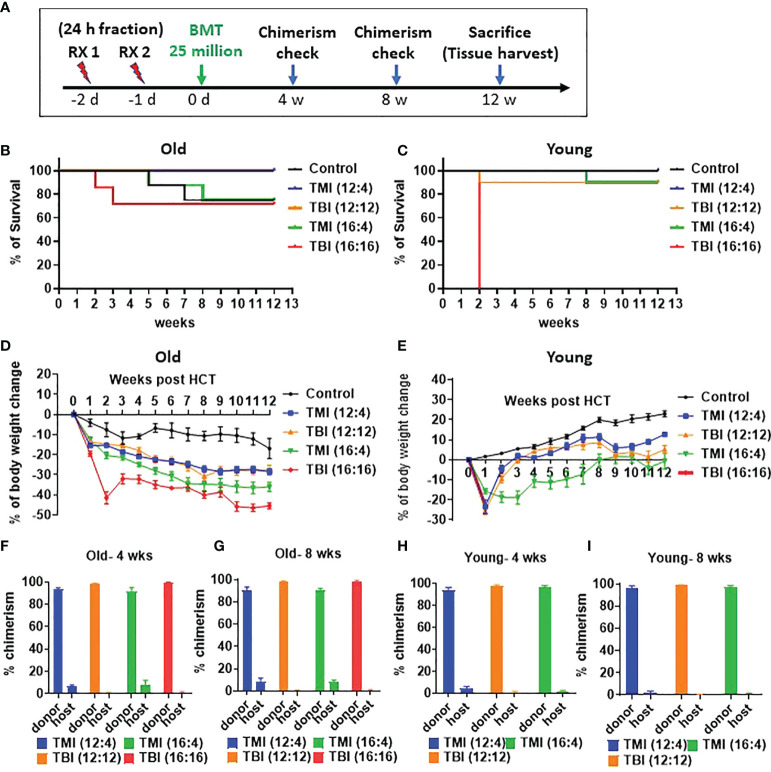
Comparison of TMI vs. TBI in survival, and donor engraftment in old and young mice. **(A)** Experiment schema. A total of 25 million of whole bone marrow cells were injected at 1 day intravenously after 2 times irradiation. **(B)** Survival rate of old mice for 12 weeks after BMT (Old control, n=8; TMI (12:4), n=10; TBI (12:12), n=10; TMI (16:4), n=8; TBI (16:16), n=7). **(C)** Survival rate of young mice for 12 weeks after BMT (Young control, n=8; TMI (12:4), n=10; TBI (12:12), n=10; TMI (16:4), n=10; TBI (16:16), n=10). **(D)** Body weight changes of old mice for 12 weeks after Rx/BMT. Mean ± SEM value. P-value are shown in **Supplementary Figure S2** and body weight changes of individual mice were shown in **Supplementary Figure 4A**. **(E)** Body weight changes of young mice for 12 weeks after Rx/BMT. Mean ± SEM value. P-value are shown in **Supplementary Figure S3** and body weight changes of individual mice were shown in **Supplementary Figure 4B**. **(F, G)** Donor engraftment of peripheral blood in old mice at 4 weeks and 8 weeks after Rx/BMT. Donor: CD45.1, Host: CD45.2. **(H, I)** Donor engraftment of peripheral blood in young mice at 4 weeks and 8 weeks after Rx/BMT. Donor: CD45.1, Host: CD45.2. Data represent the mean ± SEM, unpaired t test, Two-way ANOVA test, Log-rank Mantel-Cox test).

We evaluated whether dose escalation was feasible in both young and old mice. The study design is shown (**Figure 2A**). In both age groups, TMI (12:4) and TMI (16:4) were well tolerated and ~over 70% of mice survived 3 months post BMT (**Figures 2B, C**). Although TBI 16 Gy was well tolerated in old mice (**Figure 2B**), it was lethal in young mice (**Figure 2C**). Old mice treated with TMI (12:4) or TBI (12:12) had increased 12-week survival post BMT compared to aged-matched controls (**Figure 2B**). Despite TMI (16:4) and TBI (16:16) having similar overall 3 months survival, although not significant, the onset of mortality was much earlier when 16 Gy was given in the TBI setting (within 2-3 weeks of exposure), reminiscent of hematopoietic acute radiation syndrome (**Figure 2B**, p=0.09). This also suggests that the cause of death in 16 Gy TMI mice was likely different.

As part of toxicity analysis, body weight was measured before Rx/BMT treatment and then every week post BMT to determine % weight loss post BMT. The body weight of untreated young control mice started at a baseline average of 20g ± 2g and increased over time unlike untreated old control mice that decreased from their baseline value of 40g±5g) (**Figures 2D, E**). As expected, all irradiated mice (young and old) showed significant weight reduction compared to untreated aged-matched controls at 1-2 week after exposure (p-value in **Supplementary Figures S2**–**S4**) with little or no difference between TMI (12:4) or TBI (12:12) amongst young and old mice, respectively (**Figure 2E** p<0.05, **Figure 2D**). Old mice treated with TBI (16:16) showed accelerated weight loss at 2 weeks after TBI which was not observed in TMI (16:4) treated mice (**Figure 2D**). Young mice, starting at a much lower baseline weight than old mice (20g vs 40g), were unable to tolerate treatment with TBI (16:16) and died within a week suggesting acute GI-death (**Figure 2E**). In addition, because there is the difference of body weight between young control and old control mice, we checked the food intake in old vs. young mice. The food intake and caloric intake per day was not different between young and old mice (**Supplementary Figure S5**). This is in line with what we know about the importance of age and weight as it relates to *in vivo* radiation toxicity in mice. Further, examining donor engraftment in peripheral blood of Rx/BMT old recipient mice we observed more than 90% donor chimerism at 4 and 8 weeks irrespective of age (young or old), of treatment type (TMI or TBI at 12 Gy), or radiation dose when given as TMI (12 or 16 Gy) (**Figures 2F–I**). In essence, TMI and TBI at the 12 Gy BM dose are isoeffective with respect to donor chimerism.

### TMI causes less acute DNA damage in the gut

**Figure 3:**
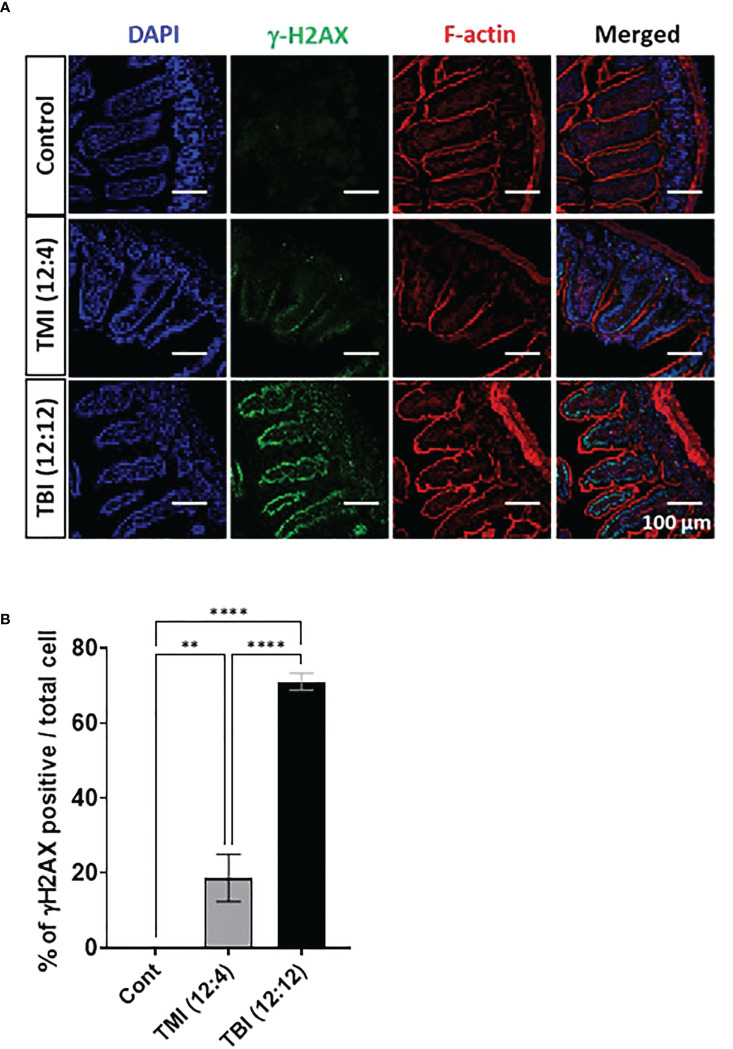
TMI reduces acute DNA damage than TBI. The mice were treated with TMI and TBI and 5 h post Rx DNA DSB was assessed by staining for γH2AX by immunofluorescence. **(A)** Immunofluorescence staining of γH2AX at 5h after irradiation. (Green = γH2AX, Red = F-actin, Blue = DAPI). (Young control, n=4; TMI (12:4), n=4; TBI (12:12), n=4) **(B)** Percentage of γH2AX positive cells/total cells. Three fields in each section and 2 different tissue sections from 4 mice in each group were used for calculations. Enlarged images are shown in **Supplementary Figure S6**. (**< 0.01, ****<0.0001, unpaired t test). Scale bar = 100 µm **(A)**.

We compared the extent of damage by measuring DNA double strand break (DSB) following TMI or TBI treatment using histone H2AX phosphorylation at Ser-139, i.e. γH2AX (17). At 5 hours after irradiation, the jejunum from TBI (12:12) treated mice showed a substantial γH2AX signal, in comparison to TMI treated gut (**Figures 3A, B** and **Supplementary Figure 6**). This suggests that at equal BM dose, TBI caused more severe DNA damage in the gut than TMI.

### TMI reduces organ damage to liver and gut compared to TBI

**Figure 4:**
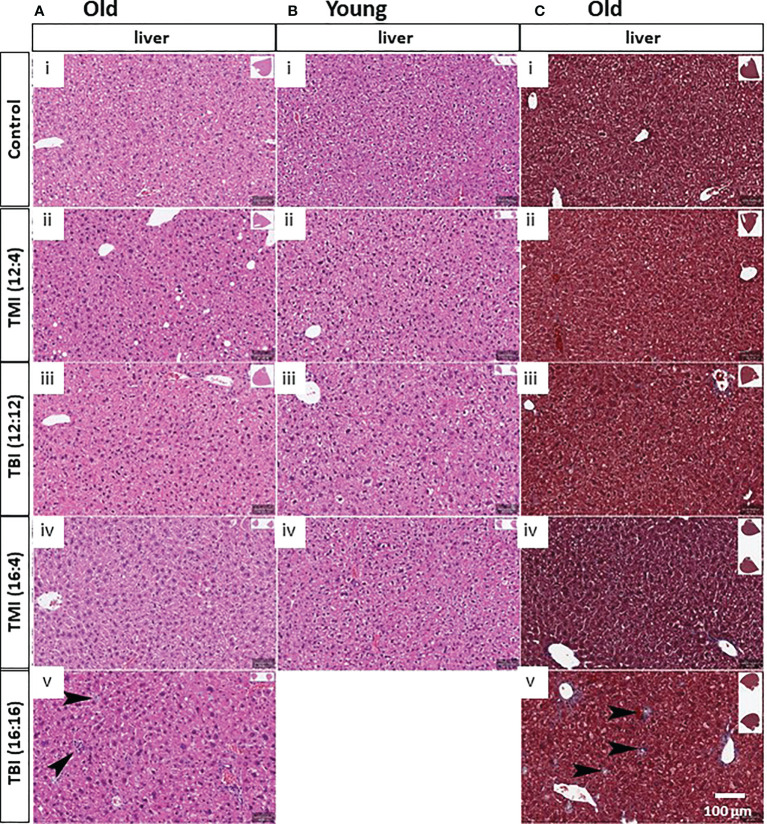
Damage of liver in TMI, TBI and untreated control in young and old mice at 12 weeks after BMT. **(A)** Liver histopathology in old mice. With TBI (16:16), there was increased portal inflammation and hepatocytes (arrows) present consistent with hepatocyte damage and turnover. Nuclear pleomorphism in the hepatocytes can be seen with increased radiation dosage. (i) Old control, (ii) TMI (12:4), (iii) TBI (12:12), (iv), TMI (16:4) and (v) TBI (16:16). **(B)** Liver histopathology in young mice. Increased nuclear pleomorphism in the hepatocytes can be seen with increased radiation dosage. (i) Young control, (ii) TMI (12:4), (iii) TBI (12:12), (iv), and TMI (16:4). **(C)** Trichrome staining in liver of old mice group. Increased fibrosis can be seen associated with the histocytes and portal inflammation. (i) Old control, (ii) TMI (12:4), (iii) TBI (12:12), (iv), TMI (16:4) and (v) TBI (16:16). Scale bar = 100 µm **(A–C)**.

We hypothesized that TMI treatment elicits less damage to normal tissues compared to TBI. There were some changes in other organs in old mice and after TMI and TBI (**Figure 4A**), such as increased portal inflammation as well as increased number of histiocytes indicative of liver damage and hepatocyte turnover (**Figure 4A**, arrows). Although, no such liver damage was observed in young mice (**Figure 4B**), but TBI (16:16) was lethal to young mice. Further, Trichrome stains of the liver also showed increased fibrosis associated with the inflammatory cells and histiocytes (**Figure 4C**, arrows). Of note, with increased radiation dose, one can appreciate the nuclear pleomorphism of the hepatocytes with increased nuclear size as well as occasional binucleated hepatocytes, indicating cellular damage (**Figure 4A**).

**Figure 5:**
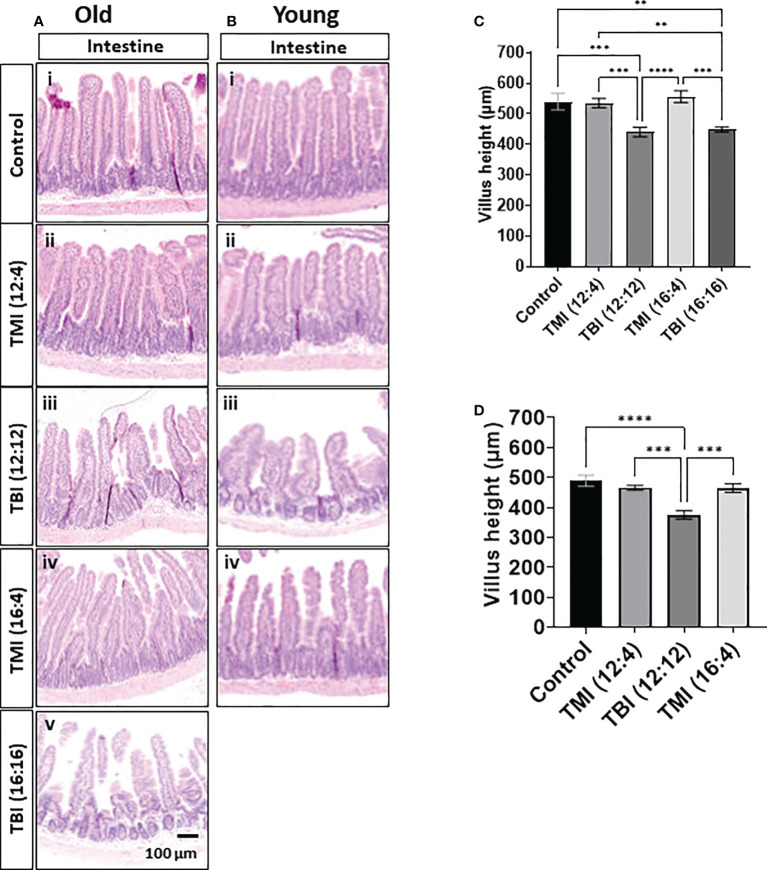
Damage of intestine in TMI, TBI, and untreated control at 12 weeks after BMT. The jejunum (~14 cm apart from the stomach) was collected and fixed for paraffin cross-section. Paraffin sections were stained with hematoxylin and eosin. **(A)** Intestinal anatomical changes in old mice. The H&E of TBI (12:4) and TBI (16:4) showed the blunt villi morphology and crypts hyperplasia compared to TMI (12:4) and TMI (16:4). (i) Old control, (ii) TMI (12:4), (iii) TBI (12:12), (iv), TMI (16:4) and (v) TBI (16:16). **(B)** Intestinal anatomical changes in young mice. (i) Young control, (ii) TMI (12:4), (iii) TBI (12:12), (iv), and TMI (16:4). The H&E of TBI (12:4) showed the blunt villi morphology and crypts hyperplasia compared to TMI (12:4) and TMI (16:4). **(C)** Measurements of villus height in old mice. (Old control n=5, TMI (12:4), n=6; TBI (12:12), n=7; TMI (16:4), n=6; TBI (16:16), n=5). Enlarged images are shown in **Supplementary Figure 7A**. **(D)** Measurements of villus height in young mice. (Young control, n=4; TMI (12:4), n=5; TBI (12:12), n=4; TMI (16:4), n=5). Enlarged images are shown in **Supplementary Figure 7B**. The villi height of 2 different gut section per each mouse was measured by ImageJ. (**< 0.01, ***<0.001, ****<0.0001, unpaired t-test). Scale bar = 100 µm **(A, B)**.

Intestine is one of the most radiosensitive organs as intestinal cells are highly proliferative. Therefore, we evaluated whether reduced organ dose in TMI protected the organ in comparison to TBI and what effect dose escalation would have on intestinal damage/repair. Histological assessment at the 3 months follow-up time point showed that TBI (12:12) and (16:16) treated old mice and TBI (12:12) treated young mice had pronounced intestinal damage in comparison to TMI (both dose levels and all age groups) (**Figures 5A, B** and **Supplementary Figure S7**). The H&E of TBI (12:4) and TBI (16:4) showed the blunt villi morphology and crypts hyperplasia compared to TMI (12:4) and TMI (16:4). TBI (both dose levels) given to old mice caused a 20% loss in villus height which was similar in young mice (~25% reduction) after TBI (lower dose) treatment. In contrast, TMI treated mice showed more healthy villus length comparable to untreated age and Sex-matched control mice (**Figures 5C, D**).

In addition, damage assessment to the lung was also carried out at 3 months post BMT. For the old mice there was mild reduction of alveolar spaces and thinning of the space walls with higher doses of radiation compared to the control. According to histopathology there were no noticeable changes in the lung, be it in the alveolar spaces or any signs of lung fibrosis both for young and old mice treated with TMI and TBI at 3 months post treatment (**Supplementary Figures S8A, B**). However, because the present treatment TMI plan covers a substantial amount of the lungs **(** **Supplementary Figure S1C** **)**, proper pathological assessment of lung damage comparing TBI and TMI is challenging and needs further studies.

### TBI causes persistent tissue damage after Rx/BMT

**Figure 6:**
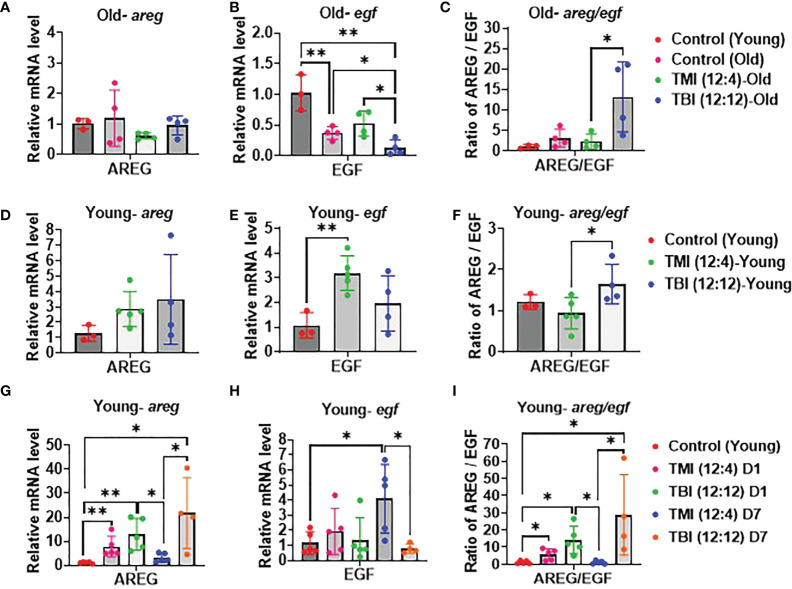
Damage and repair in TMI, TBI, and untreated control in young mice. **(A–C)** Relative mRNA level of AREG/EGF ratio, AREG and EGF expression. qPCR analysis in old mice at 12 weeks after Rx/BMT. **(D–F)** Relative mRNA level of AREG/EGF ratio, AREG and EGF expression. qPCR analysis in young mice at 12 weeks after Rx/BMT. **(G–I)** Relative mRNA level of AREG/EGF ratio, AREG and EGF expression in young mice at day 1 and day 7 after irradiation without BMT. (* <0.05, **< 0.01, unpaired t test).

Elevated AREG/EGF ratios can be a sign of unresolved tissue damage highly relevant for BMT and the onset of conditioning induced toxicity like GVHD. The relative abundance of *areg* and *egf* mRNA in the gut (jejunum) was examined by qPCR with 12 Gy as the reference dose. A significant reduction in the *areg/egf* ratio was observed when switching from TBI to TMI in both young and old mice at 12 weeks post Rx/BMT treatment (**Figures 6A–F**) with *areg* and *egf* both responding, albeit inversely. These trends in AREG and EGF were noticeable even a day or a week after TMI compared to TBI in the absence of BMT, at least in young mice (**Figures 6G–I**). Although both radiation schemes drive an acute rise in AREG, levels return to normal within one week after TMI, but not after TBI (**Figure 6I**). These results correlated with chronic GI damage identified by histopathology (see above).

## Discussion

This is the first preclinical 3D image guided bone marrow transplant study in both young and old mice for a direct, comparative evaluation of tissue damage and repair in the context of two treatment modalities, TBI and TMI. TMI is a novel, targeted, radiotherapeutic HCT pre-conditioning regimen for many hematological malignancies and disorders that is shifting the current clinical paradigm. It is based on advances in radiation dose delivery that allow us to precisely modulate doses to planned target volumes and OARs, e.g., lung, liver, GI, and kidney - a unique feature that promises to create new opportunities in Radiation Oncology. TMI can safely enhance the dose to the entire bone marrow, including “sanctuary sites” to increase leukemia cell killing, while further reducing exposure of OARs limiting acute and chronic radiation induced toxicities. Although a dose reduction to normal tissues during TMI has been reported in the clinical setting, the actual damage and/or repair in organs has yet to be characterized. In addition, the impact of dose escalation on long term toxicities in vulnerable patient populations such as pediatric and older patients have not been studied. Here we evaluated the feasibility of dose escalation in young and old mice and characterized the acute and chronic organ damage post radiation and BMT.

The principal idea of TBI is to deliver a uniform dose of ionizing radiation to the entire body, however radiosensitivity is not uniform across all organs resulting in treatment related toxicities of OAR. Despite this, TBI as conditioning for curative HCT has been used successfully for over half a century. With the advancement of chemotherapeutic agents and radiation delivering techniques, the focus has been to increase the therapeutic ratio. Currently, TMI is one of the major innovations in Radiation Oncology that shows promising results with a meaningful reduction in treatment related toxicities and better disease free and overall survival. By and large, the benefits of organ sparing using TMI in the clinic tend to be deduced from comparisons with historical TBI data (37, 38), but the actual organ damage and tissue repair processes have never been prospectively investigated, especially in the context of aging and dose escalation.

Here, image guided TMI treatment plans reduced the prescription dose to vital organs by 40-75%, while it was ~100% in TBI as shown earlier (Darren et al., 2021). Importantly, donor engraftment was equally high in TMI and TBI treated young and old mice. TBI treated mice showed persistently elevated AREG/EGF ratios, which has been linked to states of unresolved tissue damage (34). Compared to TBI, TMI not only caused less DNA damage in the GI, but AREG/EGF levels were also lower, and tissue regeneration accelerated according to GI pathophysiology. The appeal in reducing tissue damage lies in its potential to further attenuate treatment related complications like GvHD in the allogeneic transplant setting (see abstract Srideshikan et al. ASH 2022). This study suggests that myeloablation is limited by the tolerance dose of major organs, including the BM itself. In fact, the TMI model is a useful platform for future investigation into radiation tolerance limits of BM stroma as it relates to supporting full engraftment and increasing the anti-leukemic effect in older patients who have limited treatment options.

Although dose escalation using TMI was feasible in both young and old mice with no significant difference in chimerism or survival, younger mice were particularly sensitive to dose escalation using TBI. In contrast, reduced turnover and/or increased baseline cellular senescence in aged organs could have driven radio-resistance. This is in line with Hudson et al., who reported that organs of younger mice are more susceptible to radiation-induced DNA damage (39). Differences in individual’s radio-sensitivities will also relate back to germline differences in DNA repair genes as well as immune signaling genes that determine how unrepaired damage feeds into inflammatory and immune pathways and drive the chronicity of organ damage.

Organ damage in old animals tends not to be studied much, partly for financial reasons. Our current study is the first focusing on the effects of irradiation and BMT in old mice. It is known that the myeloid to lymphoid cell ratio increases as we age, suggesting a myeloid bias in older mice (40). Interestingly, we show that transplanting younger donor BM cells into a heavily irradiated, aged BM microenvironment resulted in BM cells resembling younger mice. The myeloid to lymphoid cell ratio was reduced after BMT in old mice in comparison to untreated, aged and sex matched control mice (not shown). Similarly, Guderyon et al. reported that mobilization-based transplantation of young donor hematopoietic stem cells without irradiation expands lifespan in aged mice (41). This suggests that an aged BM microenvironment can adequately support younger donor BM progenitor cells at least initially, even after irradiation. Whether or not this can be maintained indefinitely is crucial for long-term survival, and a question that needs to be addressed (42).

There are some limitations of the current study. Some vital organs that are closer to the skeletal system, such as lungs and kidney, are still exposed to high dose of prescribed doses. As our CT scan reveals, substantial lung volume of the lungs, particularly closer to the spine and posterior region receives dose as high as prescription dose. This also limited our ability for pathological evaluation of lungs after TMI. In the future, we will develop 3D sections of an entire lung to identify high dose and low dose region. Also, we recently simulated a novel sparse orthogonal collimator–based intensity modulated preclinical TMI, which will significantly reduce radiation dose to lungs and kidney and enhance dosimetric conformality to the skeletal system (43).

In conclusion, this is a novel 3D TMI preclinical BMT model that demonstrates reduced organ damage and enhanced tissue repair in TMI treated mice over TBI. The dose escalation was tolerated in old mice, suggesting a potential HCT conditioning regimen using TMI for older patients who do not qualify for myeloablative conditioning. Further studies are warranted to understand the effect of dose escalation on BME, donor engraftment, HSCs maintenance and organ damage/repair.

## Data availability statement

The original contributions presented in the study are included in the article/**Supplementary Material**. Further inquiries can be directed to the corresponding author.

## Ethics statement

The animal study was reviewed and approved by Institute of Animal Care and Use Committee (IACUC):#16064.

## Author contributions

JL, concept and experimental design, data analysis, interpretation of results and manuscript writing and editing. SH, MM and SSM, concept and experimental design, interpretation of results and manuscript writing and editing. PV, data acquisition, interpretation of results and manuscript editing. HG and AA, experimental design and TMI/TBI treatment. VVL, data acquisition, analysis, and manuscript editing. ML, Experimental design, and data analysis for food intake studies. JS, histopathological analysis, and manuscript writing and editing. SD, AP, CA, DS, DZ, GS, JL and SH, interpretation of results and manuscript writing. All authors contributed to the article and approved the submitted version.

## Funding

This work has been supported by NIH grants, 2R01CA154491 (SH) and ONCOTEST (Ghent, Belgium) (SH). The content is solely the responsibility of the authors and does not necessarily represent the official views of the National Institutes of Health.

## Acknowledgments

We would like to thank Dr Keiichi Itakura, Beckman Research Institute, Professor Emeritus, and Prof Jeffery Wong, Department of Radiation Oncology, City of Hope National medical center for scientific discussion and editing of the manuscript. We would like to thank David Kwon, senior RA, Beckman Research Institute, City of Hope National medical center for the rigorous editing of the manuscript.

## Conflict of interest

Susanta K. Hui receives honoraria from and consults for Janssen Research & Development, LLC.

The remaining authors declare that the research was conducted in the absence of any commercial or financial relationships that could be construed as a potential conflict of interest.

## Publisher’s note

All claims expressed in this article are solely those of the authors and do not necessarily represent those of their affiliated organizations, or those of the publisher, the editors and the reviewers. Any product that may be evaluated in this article, or claim that may be made by its manufacturer, is not guaranteed or endorsed by the publisher.

## References

[1] Gilson D Taylor R. Total body irradiation. report on a meeting organized by the BIR oncology committee, held at the royal institute of British architects, London, 28 November 1996. Br J Radiol (1997) 70(840):1201–3. doi: 10.1259/bjr.70.840.9505836 9505836

[2] Wong JY Filippi AR Scorsetti M Hui S Muren LP Mancosu P. Total marrow and total lymphoid irradiation in bone marrow transplantation for acute leukaemia. Lancet Oncol (2020) 21(10):e477–87. doi: 10.1016/S1470-2045(20)30342-9 33002443

[3] Copelan EA. Hematopoietic stem-cell transplantation. N Engl J Med (2006) 354(17):1813–26. doi: 10.1056/NEJMra052638 16641398

[4] Clift RA Buckner CD Appelbaum FR Bearman SI Petersen FB Fisher LD. Allogeneic marrow transplantation in patients with acute myeloid leukemia in first remission: a randomized trial of two irradiation regimens. Blood (1990) 76(9):1867–71. doi: 10.1182/blood.V76.9.1867.1867 2224134

[5] Hui SK Kapatoes J Fowler J Henderson D Olivera G Manon RR. Feasibility study of helical tomotherapy for total body or total marrow irradiation a. Med Phys (2005) 32(10):3214–24. doi: 10.1118/1.2044428 16279075

[6] Wong JY Liu A Schultheiss T Popplewell L Stein A Rosenthal J. Targeted total marrow irradiation using three-dimensional image-guided tomographic intensity-modulated radiation therapy: an alternative to standard total body irradiation. Biol Blood Marrow Transplant (2006) 12(3):306–15. doi: 10.1016/j.bbmt.2005.10.026 16503500

[7] Hui SK Verneris MR Higgins P Gerbi B Weigel B Baker SK. Helical tomotherapy targeting total bone marrow–first clinical experience at the university of Minnesota. Acta Oncol (2007) 250–5. doi: 10.1080/02841860601042449 17453378

[8] Wong JY Rosenthal J Liu A Schultheiss T Forman S Somlo G. Image-guided total-marrow irradiation using helical tomotherapy in patients with multiple myeloma and acute leukemia undergoing hematopoietic cell transplantation. Int J Radiat Oncol Biol Phys (2009) 73(1):273–9. doi: 10.1016/j.ijrobp.2008.04.071 PMC3896447 18786784

[9] Mancosu P Cozzi L Muren LP. Total marrow irradiation for hematopoietic malignancies using volumetric modulated arc therapy: A review of treatment planning studies. Phys Imaging Radiat Oncol (2019) 11:47–53. doi: 10.1016/j.phro.2019.08.001 33458277 PMC7807866

[10] Shen J Wang X Deng D Gong J Tan K Zhao H. Evaluation and improvement the safety of total marrow irradiation with helical tomotherapy using repeat failure mode and effects analysis. Radiat Oncol (2019) 14(1):1–7. doi: 10.1186/s13014-019-1433-7 31882010 PMC6935229

[11] Al Malki MM Palmer J Tsai NC Mokhtari S Hui S Tsai W. Total marrow and lymphoid irradiation as conditioning in haploidentical transplant with posttransplant cyclophosphamide. Blood Adv (2022) 6(14):4098–106. doi: 10.1182/bloodadvances.2022007264 PMC9327543 35838754

[12] Stein A. Dose escalation of total marrow and lymphoid irradiation in advanced acute leukemia. In: Total marrow irradiation. Springer Nature Switzerland (2020). p. 69–75.

[13] Sabloff M Tisseverasinghe S Babadagli ME Samant R. Total body irradiation for hematopoietic stem cell transplantation: What can we agree on? Curr Oncol (2021) 28(1):903–17. doi: 10.3390/curroncol28010089 PMC7985756 33617507

[14] Malicki J Kosicka G Stryczyńska G Wachowiak J. Cobalt 60 versus 15 MeV photons during total body irradiation: doses in the critical organs and complexicity of the procedure. Ann Transplant (2001) 6(1):18–22. 11803600

[15] Chiang Y Tsai CH Kuo SH Liu CY Yao M Li CC. Reduced incidence of interstitial pneumonitis after allogeneic hematopoietic stem cell transplantation using a modified technique of total body irradiation. Sci Rep (2016) 6:36730. doi: 10.1038/srep36730 27830767 PMC5103225

[16] Ujaimi RK Isfahanian N Russa DJL Samant R Bredeson C Genest P. Pulmonary toxicity following total body irradiation for acute lymphoblastic leukaemia: The Ottawa hospital cancer centre (TOHCC) experience. J Radiother Pract (2015) 15(1):54–60. doi: 10.1017/S1460396915000497

[17] Hill-Kayser CE Plastaras JP Tochner Z Glatstein E. TBI during BM and SCT: review of the past, discussion of the present and consideration of future directions. Bone Marrow Transplant (2011) 46(4):475–84. doi: 10.1038/bmt.2010.280 21113184

[18] Felice DEF Grapulin L Musio D Pomponi J Cinzia DIF Iori AP. Treatment complications and long-term outcomes of total body irradiation in patients with acute lymphoblastic leukemia: A single institute experience. Anticancer Res (2016) 36(9):4859–64. doi: 10.21873/anticanres.11049 27630341

[19] Thomas ED Clift RA Hersman J Sanders JE Stewart P Buckner CD. Marrow transplantation for acute nonlymphoblastic leukemic in first remission using fractionated or single-dose irradiation. Int J Radiat Oncol Biol Phys (1982) 8(5):817–21. doi: 10.1016/0360-3016(82)90083-9 7050046

[20] Shank B Chu FC Dinsmore R Kapoor N Kirkpatrick D Teitelbaum H. Hyperfractionated total body irradiation for bone marrow transplantation. results in seventy leukemia patients with allogeneic transplants. Int J Radiat Oncol Biol Phys (1983) 9(11):1607–11. doi: 10.1016/0360-3016(83)90412-1 6358154

[21] Deeg HJ Sullivan KM Buckner CD Storb R Appelbaum FR Clift RA. Marrow transplantation for acute nonlymphoblastic leukemia in first remission: toxicity and long-term follow-up of patients conditioned with single dose or fractionated total body irradiation. Bone Marrow Transplant (1986) 1(2):151–7. 3332129

[22] Gopal R Ha CS Tucker SL Khouri IF Giralt SA Gajewski JL. Comparison of two total body irradiation fractionation regimens with respect to acute and late pulmonary toxicity. Cancer (2001) 92(7):1949–58. doi: 10.1002/1097-0142(20011001)92:7<1949::AID-CNCR1714>3.0.CO;2-1 11745270

[23] Giebel S Miszczyk L Slosarek K Moukhtari L Ciceri F Esteve J. Extreme heterogeneity of myeloablative total body irradiation techniques in clinical practice: a survey of the acute leukemia working party of the European group for blood and marrow transplantation. Cancer (2014) 120(17):2760–5. doi: 10.1002/cncr.28768 24804873

[24] Cheng JC Schultheiss TE Wong JY. Impact of drug therapy, radiation dose, and dose rate on renal toxicity following bone marrow transplantation. Int J Radiat Oncol Biol Phys (2008) 71(5):1436–43. doi: 10.1016/j.ijrobp.2007.12.009 18355974

[25] Altschuler C Resbeut M Blaise D Maraninchi D Stoppa AM Lagrange JL. Fractionated total body irradiation and bone marrow transplantation in acute lymphoblastic leukemia. Int J Radiat Oncol Biol Phys (1990) 19(5):1151–4. doi: 10.1016/0360-3016(90)90220-E 2254105

[26] Schneider RA Schultze J Jensen JM Hebbinghaus D Galalae RM. Long-term outcome after static intensity-modulated total body radiotherapy using compensators stratified by pediatric and adult cohorts. Int J Radiat Oncol Biol Phys (2008) 70(1):194–202. doi: 10.1016/j.ijrobp.2007.05.035 17869024

[27] Wong JYC Filippi AR Dabaja BS Yahalom J Specht L. Total body irradiation: Guidelines from the international lymphoma radiation oncology group (ILROG). Int J Radiat Oncol Biol Phys (2018) 101(3):521–9. doi: 10.1016/j.ijrobp.2018.04.071 29893272

[28] Hui S Takahashi Y Holtan SG Azimi R Seelig D Yagi M. Early assessment of dosimetric and biological differences of total marrow irradiation versus total body irradiation in rodents. Radiother Oncol (2017) 124(3):468–74. doi: 10.1016/j.radonc.2017.07.018 PMC5624834 28778346

[29] Zuro D Madabushi SS Brooks J Chen BT Goud J Salhotra A. First multimodal, three-dimensional, image-guided total marrow irradiation model for preclinical bone marrow transplantation studies. Int J Radiat Oncol Biol Phys (2021) 111(3):671–83. doi: 10.1016/j.ijrobp.2021.06.001 PMC8565655 34119592

[30] Qi Y Operario DJ Georas SN Mosmann TR. The acute environment, rather than T cell subset pre-commitment, regulates expression of the human T cell cytokine amphiregulin. PloS One (2012) 7(6):e39072. doi: 10.1371/journal.pone.0039072 22720031 PMC3375254

[31] Berasain C Avila MA. Amphiregulin. Semin Cell Dev Biol (2014) 28:31–41. doi: 10.1016/j.semcdb.2014.01.005 24463227

[32] Zaiss DMW Gause WC Osborne LC Artis D. Emerging functions of amphiregulin in orchestrating immunity, inflammation, and tissue repair. Immunity (2015) 42(2):216–26. doi: 10.1016/j.immuni.2015.01.020 PMC4792035 25692699

[33] Holtan SG Shabaneh A Betts BC Rashidi A MacMillan ML Ustun C. Stress responses, M2 macrophages, and a distinct microbial signature in fatal intestinal acute graft-versus-host disease. JCI Insight (2019) 5(17):e129762. doi: 10.1172/jci.insight.129762 31393854 PMC6777917

[34] Atkins SLP DeFor TE MacMillan ML Turcotte L Rashidi A Weisdorf DJ. Elevated AREG/EGF ratio prior to transplantation is associated with pre-transplant clostridium difficile infection, unresolved tissue damage, and poorer overall survival. Blood (2018) 132:3353. doi: 10.1182/blood-2018-99-115686

[35] Ceafalan LC Manole E Tanase CP Codrici E Mihai S Gonzalez A. Interstitial outburst of angiogenic factors during skeletal muscle regeneration after acute mechanical trauma. Anatomical Rec (2015) 298(11):1864–79. doi: 10.1002/ar.23254 26260512

[36] Holtan SG Khera N Levine JE Chai X Storer B Liu HD. Late acute graft-versus-host disease: a prospective analysis of clinical outcomes and circulating angiogenic factors. Blood J Am Soc Hematol (2016) 128(19):2350–8. doi: 10.1182/blood-2015-09-669846 PMC5106113 27625357

[37] Haraldsson A Wichert S Engström PE Lenhoff S Turkiewicz D Warsi S. Organ sparing total marrow irradiation compared to total body irradiation prior to allogeneic stem cell transplantation. Eur J Haematol (2021) 107(4):393–407. doi: 10.1111/ejh.13675 34107104

[38] Shinde A Yang D Frankel P Liu A Han C Del Vecchio B. Radiation-related toxicities using organ sparing total marrow irradiation transplant conditioning regimens. Int J Radiat Oncol Biol Phys (2019) 105(5):1025–33. doi: 10.1016/j.ijrobp.2019.08.010 31421151

[39] Hudson D Kovalchuk I Koturbash I Kolb B Martin OA Kovalchuk O. Induction and persistence of radiation-induced DNA damage is more pronounced in young animals than in old animals. Aging (Albany NY) (2011) 3(6):609. doi: 10.18632/aging.100340 21685513 PMC3164369

[40] Morrison SJ Wandycz AM Akashi K Globerson A Weissman IL. The aging of hematopoietic stem cells. Nat Med (1996) 2(9):1011–6. doi: 10.1038/nm0996-1011 8782459

[41] Guderyon MJ Chen C Bhattacharjee A Ge G Fernandez RA Gelfond JA. Mobilization-based transplantation of young-donor hematopoietic stem cells extends lifespan in mice. Aging Cell (2020) 19(3):e13110. doi: 10.1111/acel.13110 32012439 PMC7059148

[42] Dorshkind K Höfer T Montecino-Rodriguez E Pioli PD Rodewald HR. Do haematopoietic stem cells age? Nat Rev Immunol (2020) 20(3):196–202. doi: 10.1038/s41577-019-0236-2 31740804 PMC7879798

[43] Abdelhamid AMH Jiang L Zuro D Liu A Madabushi SS Ghimire H. Feasibility of a novel sparse orthogonal collimator-based preclinical total marrow irradiation for enhanced dosimetric conformality. Front Oncol (2022) 12:941814. doi: 10.3389/fonc.2022.941814 35924145 PMC9339640

