# Supplementary material for: Total marrow irradiation reduces organ damage and enhances tissue repair with the potential to increase the targeted dose of bone marrow in both young and old mice
Source: Front Oncol. 2022 Nov 10;12:1045016. doi: 10.3389/fonc.2022.1045016 (PMC9686437; doi:10.3389/fonc.2022.1045016)

Supplement figure legends

Supplement figure S1. TBI and TMI radiation treatment planning beam arrangement. (**A**) TMI planning beams arrangement in sagittal and coronal view. (**B**) TBI planning beams arrangement in sagittal and coronal view. **(C)** TMI planning beams arrangement in cross-sectional view at the level of lungs.

Supplement figure S2. Body weight change and p-value in old mice. Mice body weigh was measured at indicated time points. Data represents the mean ± SEM (* <0.05, **< 0.01, *** <0.001, **** <0.0001, Two-way ANOVA test).

Supplement figure S3. Body weight change and p-value in young mice. Mice body weigh was measured at indicated time points. Data represents the mean ± SEM (* <0.05, **< 0.01, *** <0.001, **** <0.0001, Two-way ANOVA test).

Supplement figure S4. Individual body weight change. Mice body weigh was measured at indicated time points. (**A**) Individual body weight changes of old mice for 12 weeks after Rx/BMT. (Old control, n=8; TMI (12:4), n=10; TBI (12:12), n=10; TMI (16:4), n=8; TBI (16:16), n=7). (**B**) Individual body weight changes of young mice for 12 weeks after Rx/BMT. (Young control, n=8; TMI (12:4), n=10; TBI (12:12), n=10; TMI (16:4), n=10; TBI (16:16), n=10).

Supplement figure S5. Food intake and caloric intake per day between old and young mice. (**A**) Food intake per day (g). Food intake (Low-fat chow diet, Picolab, #5053) was measured every 2 days for 2 weeks. (**B**) Caloric intake per day (Kcal). Gross energy of Low-fat chow diet (Picolab, #5053) is Kcal/g = 4.11. Daily caloric intake= (food intake in gm/mouse/day) x 4.11.

Supplement figure S6. Representative enlarged images of γH2AX staining in gut at 5h after Rx. The 1cm of jejunum (~14 cm apart from stomach) was fixed in 10% NBF and transferred into 30% sucrose in PBS and cut by cryosection. DNA damage was assessed by radiation induced DSB by immunofluorescence using anti-γH2AX antibody. TBI treated mice show increased γH2AX staining in comparison to TMI. (Green = γH2AX, Red = F-actin, Blue = DAPI). Scale bar= 200μm.

Supplement figure S7. Intestine histology at 12 weeks after Rx/BMT. Representative enlarged images of gut cross section. 1cm Jejunum (~14 cm apart from stomach) was fixed in 10% NBF and cut by paraffin cross section. (**A**) Intestinal anatomical changes in old mice. (i) Old control, (ii) TMI (12:4), (iii) TBI (12:12), (iv), TMI (16:4) and (v) TBI (16:16). (**B**) Intestinal anatomical changes in young mice. (i) Young control, (ii) TMI (12:4), (iii) TBI (12:12), (iv), and TMI (16:4). Scale bar = 1 mm (**A, B**).

Supplement figure S8. H&E-stained sections of lung from TMI and TBI treated mice. (**A**) Lung histology of old mice. (i) control, (ii) TBI (12:12), and (iii) TBI (16:16). (**B**) Lung histology of young mice. (i) control, and (ii) TBI (12:12). Scale bar = 100 μm (**A, B**).

Supplement Table 1. 3D-Preclinical TBI and TMI beam arrangements for the radiation treatment planning. Radiation beam layout of TBI and TMI by regions (beam size, isocenter location, normalization point), respectively. (**A**) For TBI planning, mouse CBCT scans were divided into 3 regions for treatment optimization. For each one of those three regions, parallel opposed beams with beam size of 40 mm square collimator were used to create a homogenized dose within the center of the beams. (**B**) For TMI planning, Mouse CT scans were divided into 7 regions for treatment optimization. Beam sizes were varied (40x40mm to 5mm square or circle) in different regions using different collimator settings.

Supplement Table 1

A

TBI Beam layout by region			
**Region**	**Beam Size**	**Isocenter**	**Normalization**
**Skull**	40 mm square	Upper Brain	Brain
**Cervical, chest, and upper Abdomen**	40 mm square	Lower Chest	Upper abdomen
**Lower abdomen and pelvis**	40 mm square	Secrum	Pelvis

B

TMI Beam layout by region			
**Region**	Beam Size	Isocenter	Normalization
**Skull & Cspine**	40 mm square	Center of brain	Brain
**C to T spine junction**	5 mm circle	above scapula	Upper abdomen
**Tspine & Lspine**	20 mm square	Above the spine so the edge of the beam covers the spine	Above the spine body
**Pelvis**	20 mm square	Above the pelvis so the edge of the beam covers the pelvic girdle	Above the pelvic crest
**Femurs & legs**	20 mm square	Below the femur	BM of femur
**Spleen**	10 mm square	Within the spleen	Spleen Tissue
**Shoulders**	10 mm square	Over the elbow joint	BM of shoulder

Supplement Figure S1

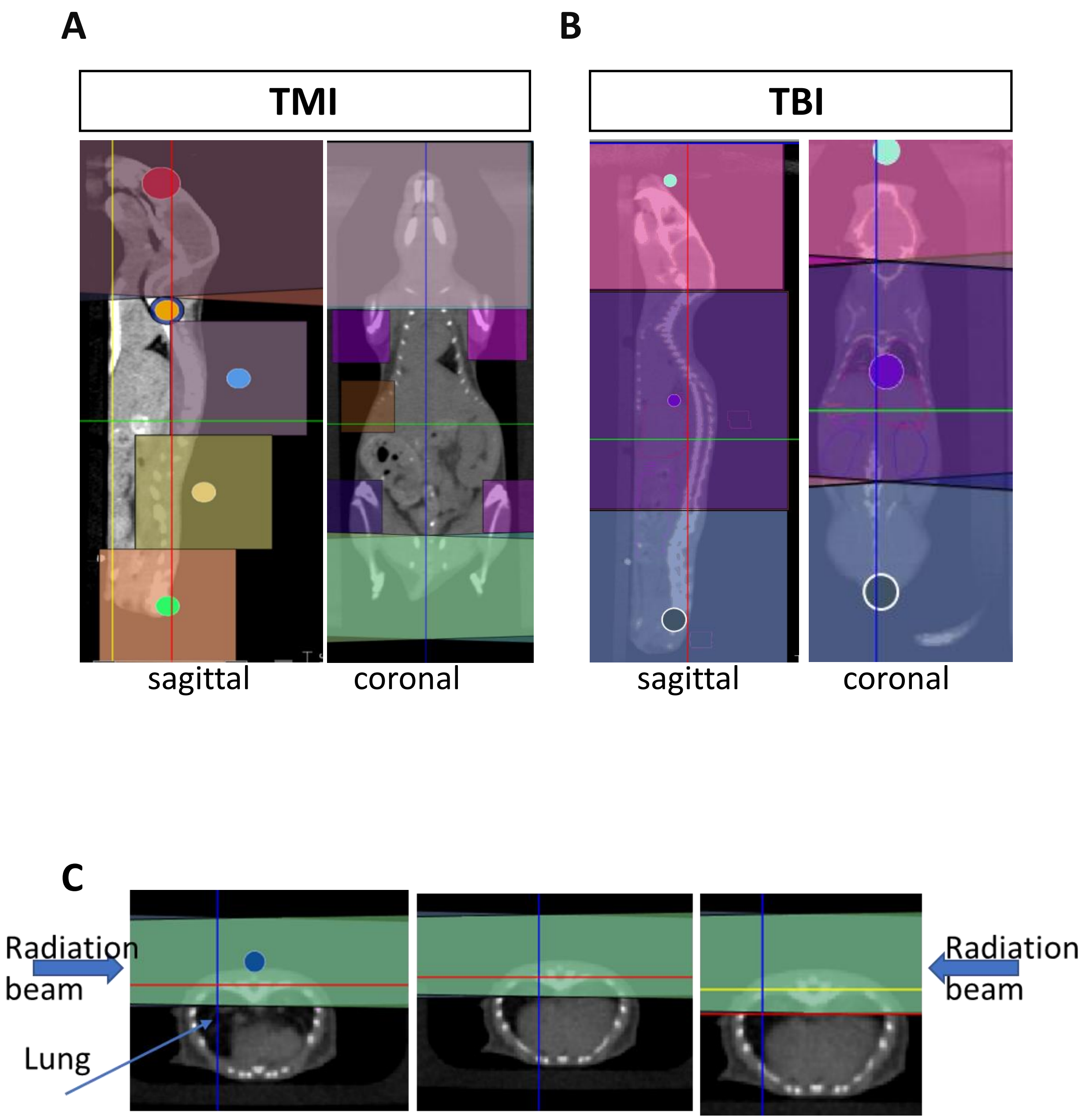

Supplement Figure S2

Old

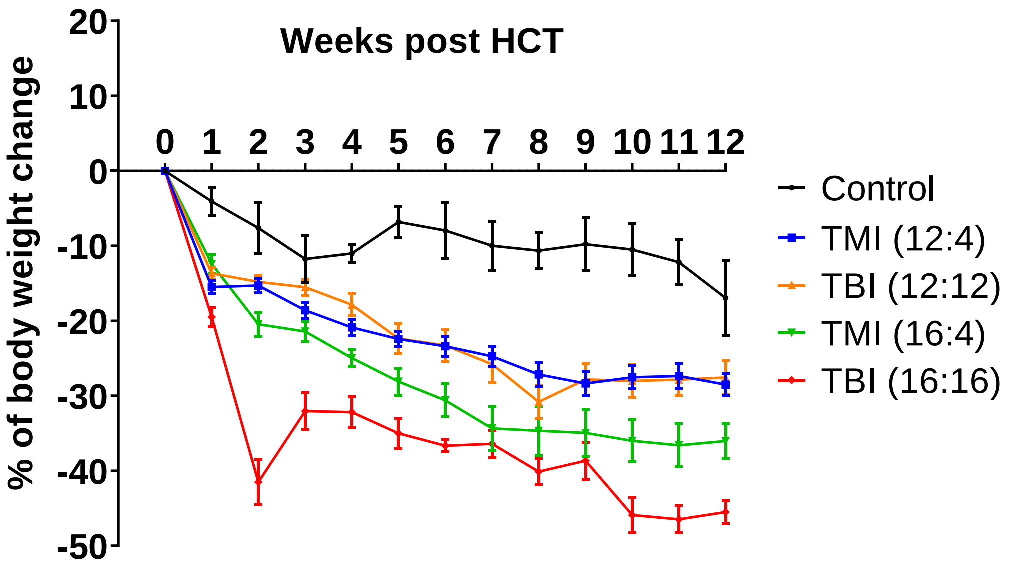

Weeks	0	1	2	3	4	5	6	7	8	9	10	11	12
Aged cont vs. TMI (12:4)	ns	****	**	**	***	****	****	****	****	****	****	****	***
Aged cont vs. TBI (12:12)	ns	***	**	ns	*	****	****	****	****	****	****	****	***
Aged cont vs. TMI (16:4)	ns	**	****	***	****	****	****	****	****	****	****	****	****
Aged cont vs. TBI (16:16)	ns	****	****	****	****	****	****	****	****	****	****	****	****
TMI (12:4) vs. TBI (12:12)	ns	ns	ns	ns	ns	ns	ns	ns	ns	ns	ns	ns	ns
TMI (12:4) vs. TMI (16:4)	ns	ns	*	ns	ns	*	**	***	**	*	**	**	**
TMI (12:4) vs. TBI (16:16)	ns	ns	****	****	***	****	****	***	****	***	****	****	****
TBI (12:12) vs. TMI (16:4)	ns	ns	*	*	**	*	**	**	ns	*	**	**	**
TBI (12:12) vs. TBI (16:16)	ns	*	****	****	****	****	****	***	**	***	****	****	****
TMI (16:4) vs. TBI (16:16)	ns	*	****	***	*	*	ns	ns	ns	ns	**	**	**

Supplement Figure S3

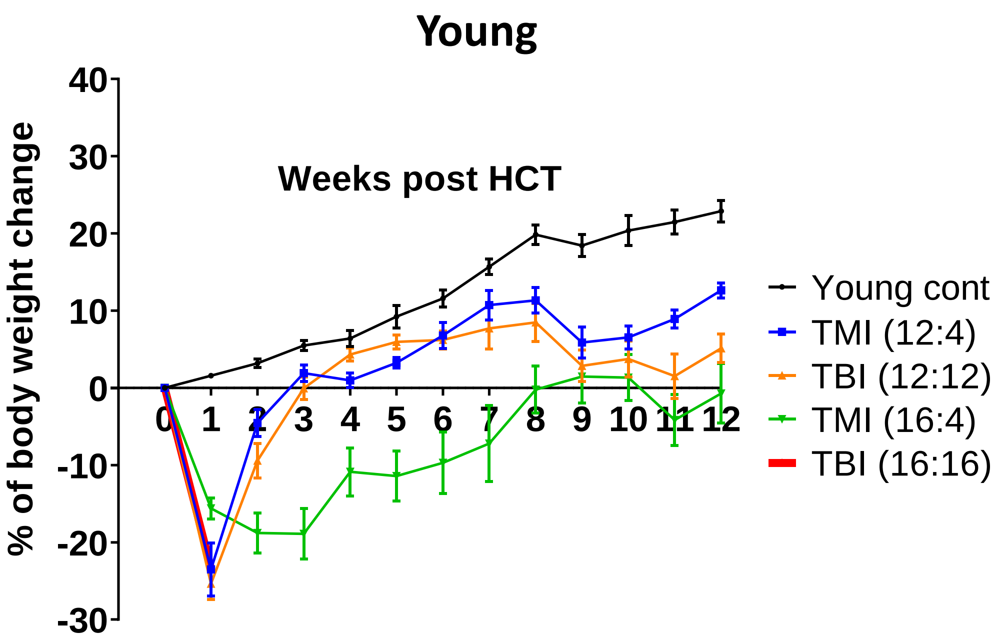

Weeks	0	1	2	3	4	5	6	7	8	9	10	11	12
**Young cont vs. TMI (12:4)**	ns	****	*	ns	ns	ns	ns	ns	*	***	****	***	**
**Young cont vs. TBI (12:12)**	ns	****	***	ns	ns	ns	ns	*	***	****	****	****	****
**Young cont vs. TMI (16:4)**	ns	****	****	****	****	****	****	****	****	****	****	****	****
**TMI (12:4) vs. TBI (12:12)**	ns	ns	ns	ns	ns	ns	ns	ns	ns	ns	ns	*	*
**TMI (12:4) vs. TMI (16:4)**	ns	**	****	****	****	****	****	****	***	ns	ns	****	****
**TBI (12:12) vs. TMI (16:4)**	ns	**	**	****	****	****	****	****	**	ns	ns	ns	ns

Supplement Figure S4

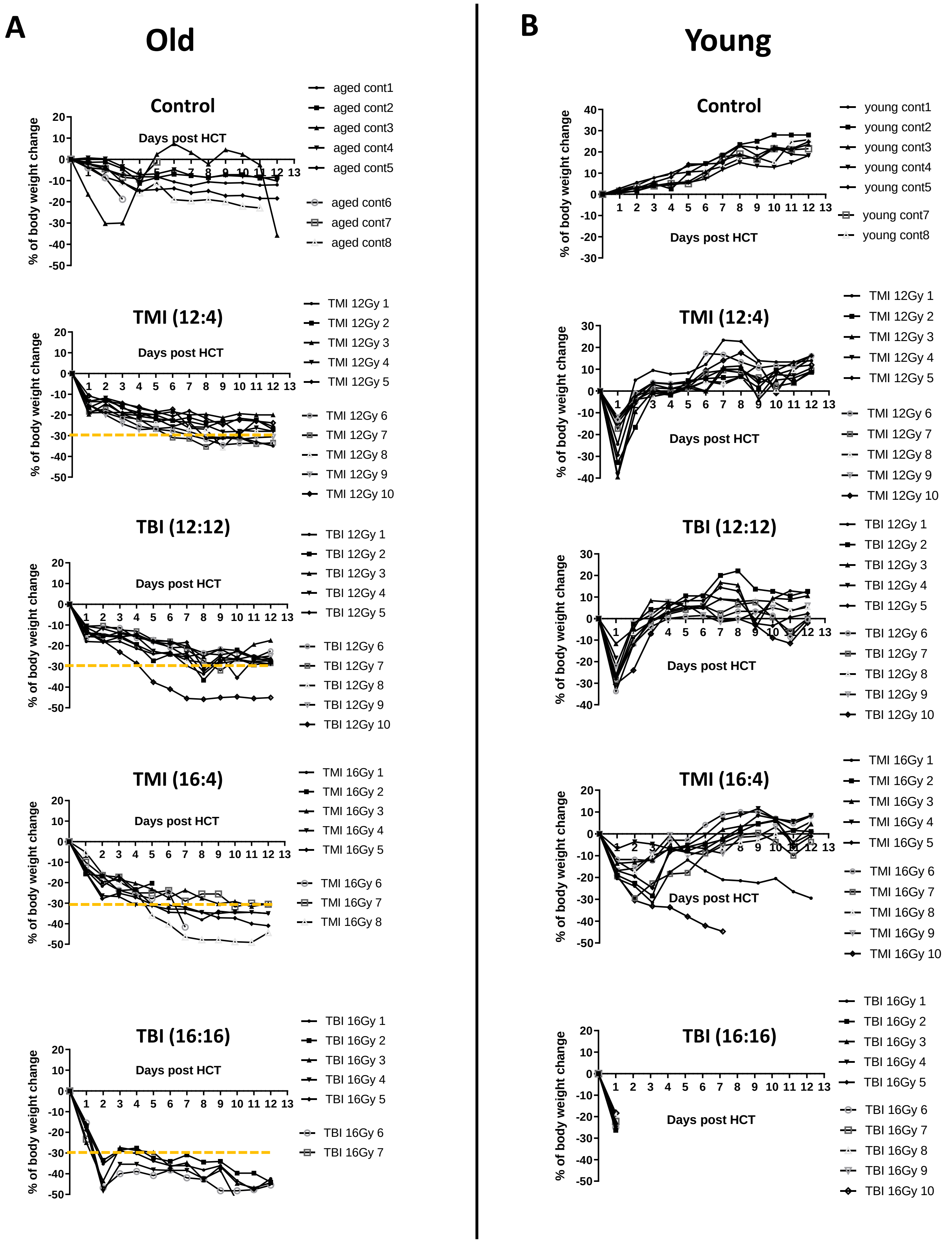

Supplement Figure S5

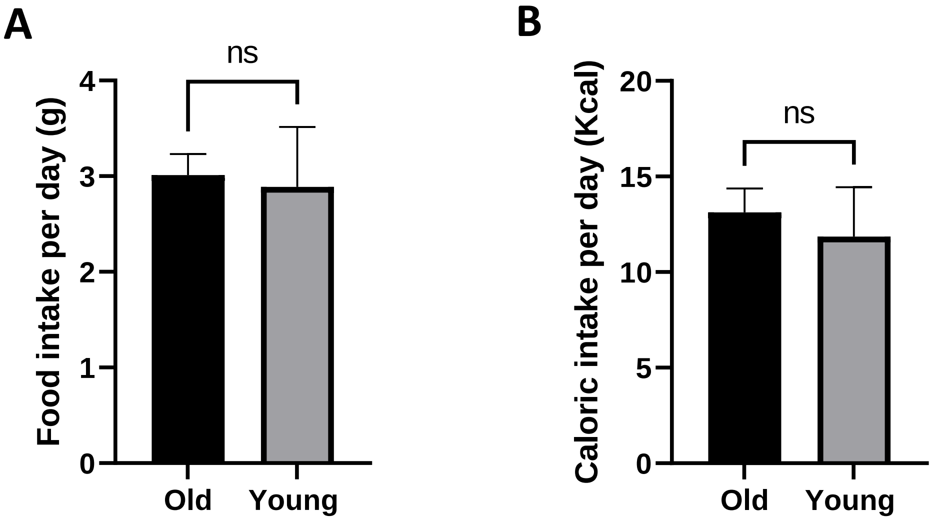

Food intake per day (g) and caloric intake per day (Kcal)

Supplement Figure S6

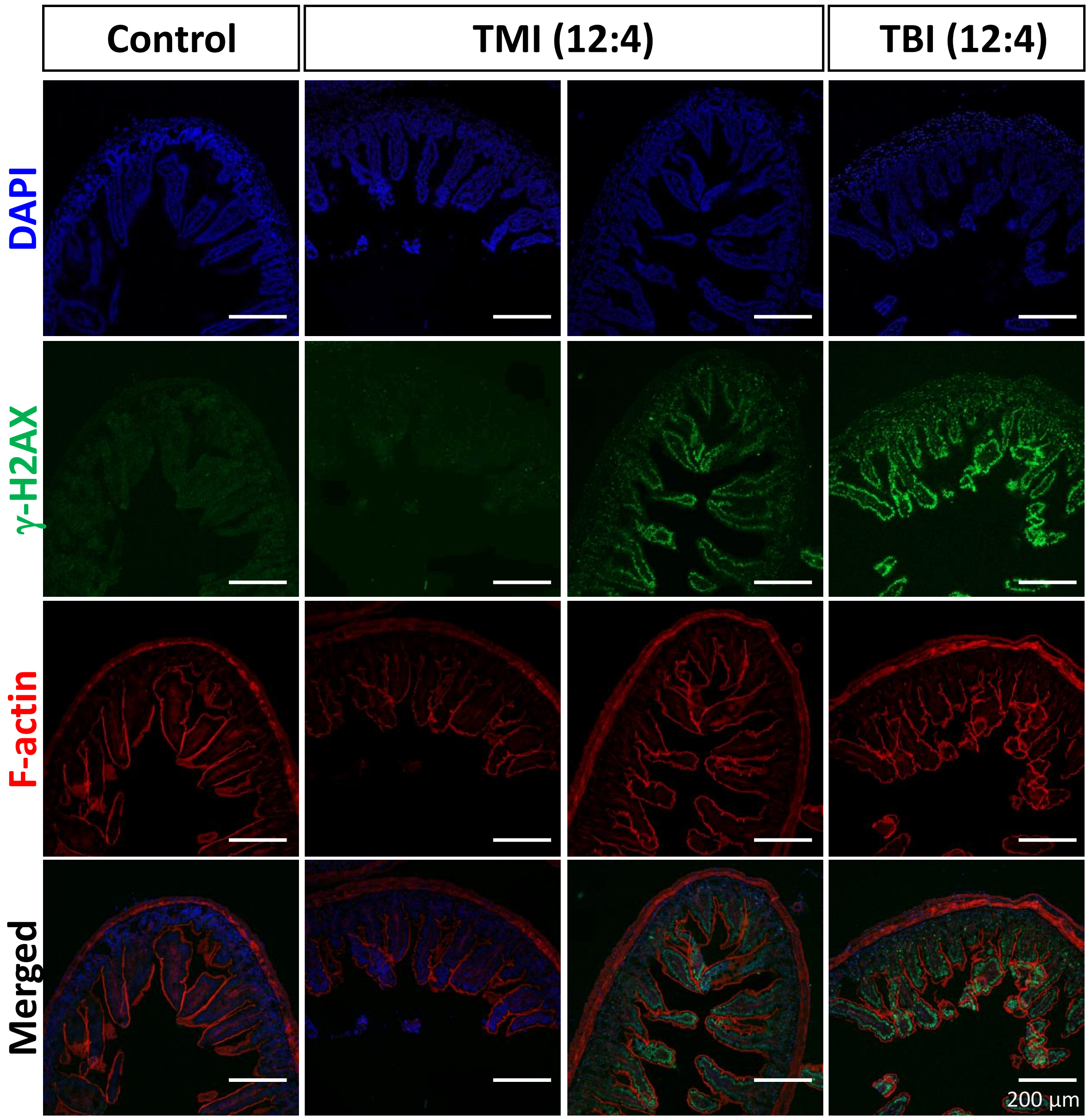

Supplement Figure S7

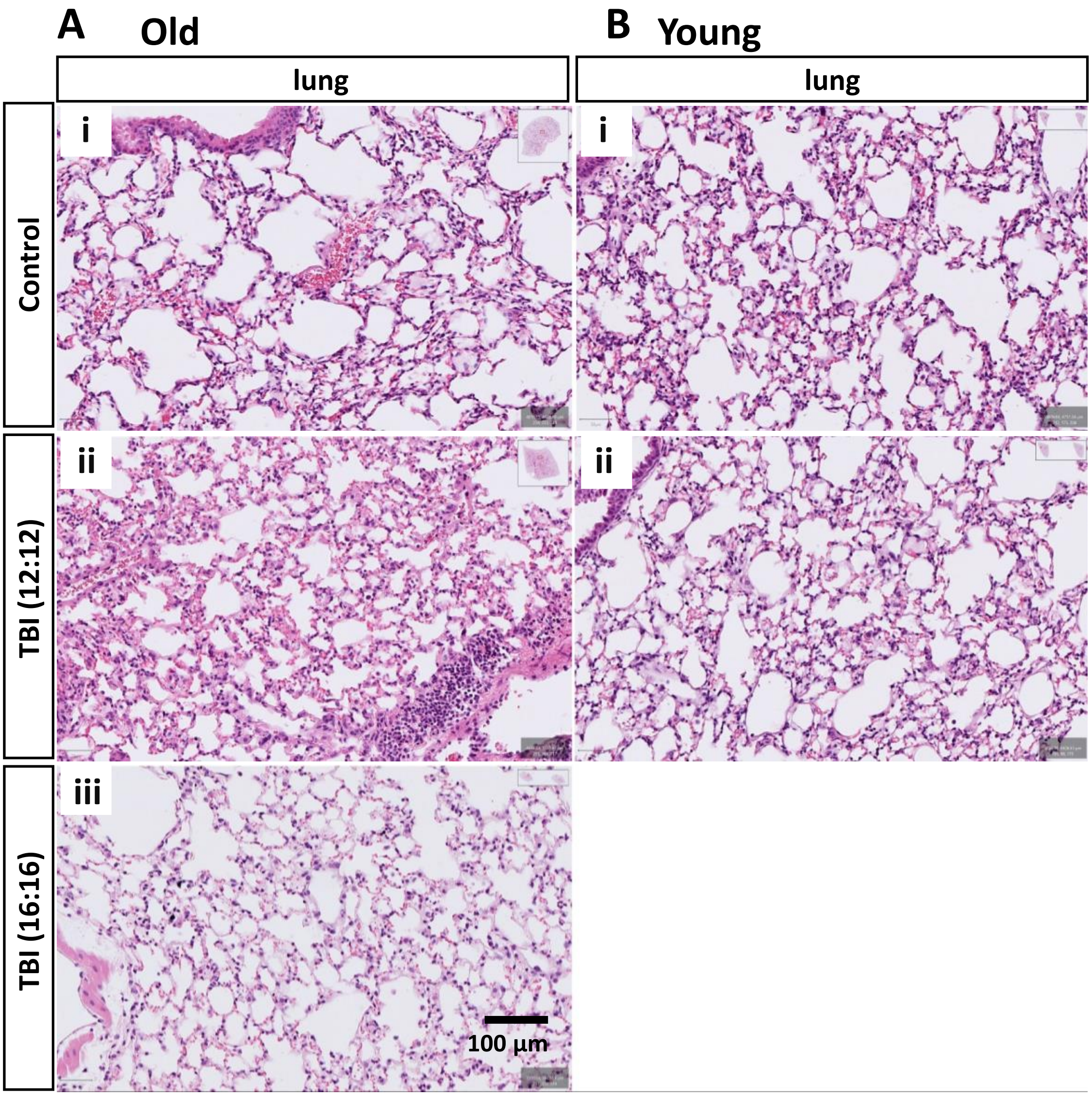

Supplement Figure S8

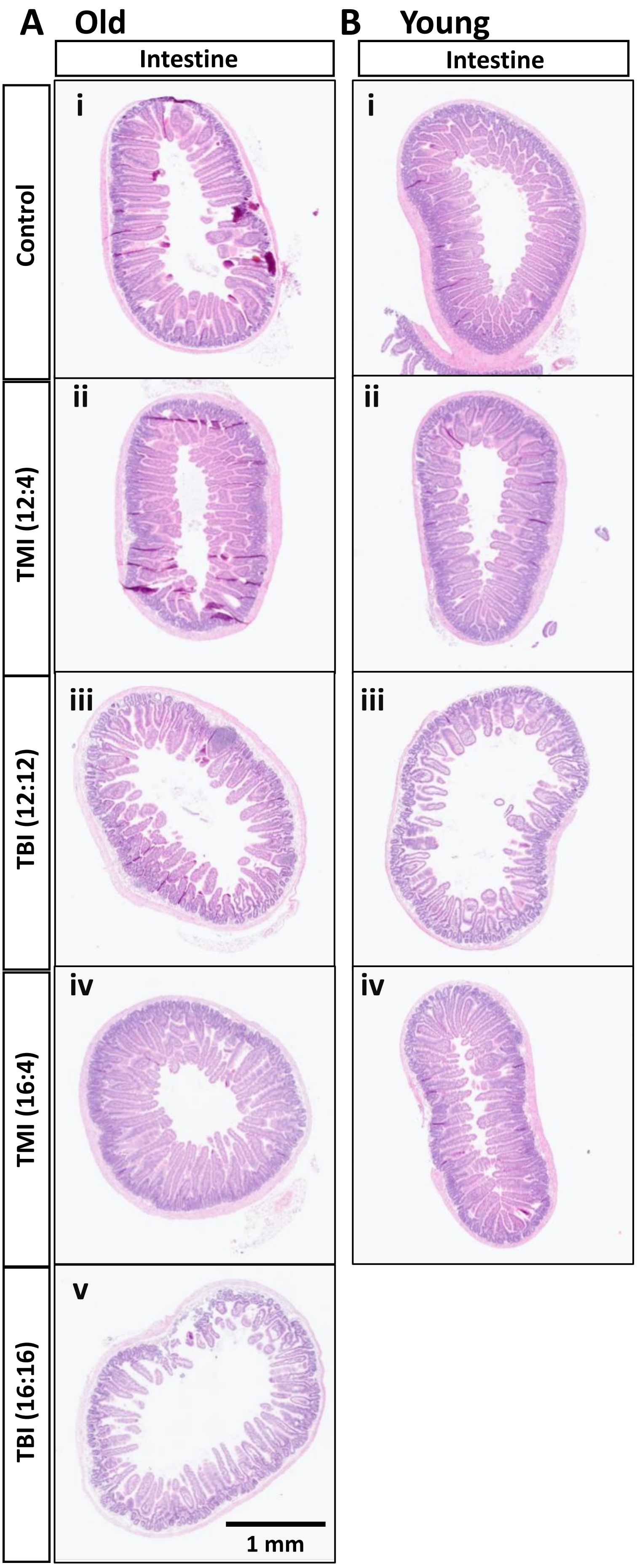

Supplement: Supplementary file 1 [file DataSheet_1.pdf]
